# A System of Oral Surgery

**Published:** 1873-01

**Authors:** 


*A System of Oral Surgery. Being a consideration of the Diseases and Surgery of the Mouth, Jaws, and associate parts.* By JAMES GARRETSON, M. D., D. D. S. Philadelphia: J. B. Lippincott & Co., 1873. Buffalo: H. H. Otis.

The work before us comprises over one thousand pages, and treats of the diseases of the mouth and jaws in all their variety. Most of the topics are illustrated by cases drawn from the experience of the writer and others. The cases are all well written, and ample directions given for treatment of the various forms of disease which affect these parts.

The work is exhaustive in scope, and for the dentist or surgical practitioner contains much of value.

